# Impact of a Community-Based Weight Loss Program on Renal Function

**DOI:** 10.7759/cureus.8101

**Published:** 2020-05-13

**Authors:** Tiffany E Schwasinger-Schmidt, Georges Elhomsy, Bobbie G Paull-Forney

**Affiliations:** 1 Internal Medicine, Kansas University School of Medicine-Wichita, Wichita, USA; 2 Weight Management Clinic, Ascension Via Christi Hospitals, Wichita, USA

**Keywords:** obesity, kidney function, weight loss, lifestyle intervention, improved ckd stage, behavioral change in weight loss, nutrition and metabolism, diabetes mellitus, hypertension, community based weight loss intervention

## Abstract

Introduction

Obesity is associated with increased morbidity and mortality and is an independent risk factor for the development and progression of chronic kidney disease (CKD). This study investigated the effect of a community-based, lifestyle-focused, weight-loss intervention on renal function among participants at baseline following 12 weeks of therapy.

Methods

A retrospective analysis of adults enrolled in a weight management program from 2009 to 2014 was conducted. Participants consumed at least 800 kilocalories per day in meal replacements, attended weekly behavioral education classes, and expended approximately 300 kilocalories per day in physical activity. The primary outcome was the association of weight loss and changes in glomerular filtration rate (GFR). Secondary outcomes included changes in blood sugar levels, lipid parameters, blood pressure, and the use of medication for hypertension and diabetes mellitus.

Results

Of the 71 participants, 63.4% were female, the average weight was 289 pounds, the average body mass index (BMI) of 53, and baseline GFR 47 ml/min/1.73m^2^. Following 12 weeks of the intervention, 76.1% of participants improved in CKD stage, 22.4% remained within the same stage, and 1.5% progressed to a higher stage (3A to 3B). Analysis revealed a correlation between weight loss and improved GFR (p=0.0006). Improvements were noted in blood sugar levels, blood pressure, and lipids (p<0.05). Medications were reduced in 61.8% of participants for hypertension and 83.3% for diabetes.

Conclusions

A significant correlation was observed between weight loss and improved renal function, with most participants improving in CKD stage. Participants also improved in markers of chronic disease and required fewer medications. When controlling for both diabetes and hypertension, the effect of improved renal function persisted.

## Introduction

World obesity rates have tripled since 1975, and an estimated 1.9 billion individuals are obese with a body mass index (BMI) of greater than 30 and an additional 650 million are overweight (BMI>25) [1]. In the United States, the age-adjusted prevalence of obesity in adults is 42.4%, which equates to approximately 139 million Americans [2]. Obesity is associated with increased morbidity and mortality and is linked to multiple chronic medical conditions including hypertension, diabetes mellitus, obstructive sleep apnea, and non-alcoholic fatty liver disease [1, 3]. The focus of obesity research has centered on cardiovascular outcomes, especially coronary artery, cerebrovascular, and peripheral vascular disease incidence, morbidity, and mortality, along with the development of dilated cardiomyopathy. More recently, attention has also been given to the effects of obesity on renal outcomes, which may be even more significant than the cardiovascular contributions to the etiology of obesity-associated morbidity and mortality. The effects of obesity on the kidney have both a direct impact through the development of renal failure, and indirect impacts through the effects of the renal system on cardiovascular outcomes [4].

Obesity is a strong independent risk factor for the development and progression of chronic kidney disease (CKD) [3]. The Prevention of Renal and Vascular End Stage Disease (PREVEND) study of 7,676 obese and overweight individuals without a baseline diagnosis of diabetes showed an elevated risk of developing CKD and decreased renal filtration correlated with obesity and central adiposity [5]. Additional studies supporting this association include the Nationwide US Veteran Administration Cohort Study of 453,946 veterans with BMIs ranging from <20 to >50. This study found that moderate to severe obesity was associated with worse renal outcomes [6]. The Kaiser Permanente Northern California study of 320,252 adults with and without baseline CKD showed a linear increase in the risk of end-stage renal disease (ESRD) with increasing weight. The relative risk was 1.87 for overweight individuals (BMI 25-29.9), 3.57 for participants with class I obesity (BMI 30-34.9), 6.12 for those with class II obesity (BMI 35-39.9) and 7.07 for individuals with a BMI >40 [7].

Rates of CKD have paralleled the rising prevalence of obesity worldwide. In the United States, 15% of adults, approximately 37 million people, are estimated to have CKD with 20,000 new cases of ESRD diagnosed per year [8]. The number of individuals with CKD attributed to obesity is difficult to estimate as kidney disease is often asymptomatic, and half the patients with CKD remain undiagnosed until they have advanced disease [89]. Mortality rates due to CKD are growing with approximately 105,351 deaths per year, which is a 50 percent increase in mortality rate since 1996 [8].

Elevations in BMI are associated with the development of proteinuria, glomerular hyperfiltration, and damage to the renal tubules [10]. The predominant mechanism underlying renal dysfunction in obese patients is believed to involve chronic inflammation and does not appear to require baseline chronic kidney disease [3, 11-15]. In population-based studies, patients with class II or class III obesity had greater declines in renal function and increased prevalence of ESRD compared to non-obese patients [12]. Obesity has also been associated with an increased risk of malignancies, including renal cell carcinoma [12-13]. The effect of obesity on the renal system has a disproportionate impact on the cardiovascular system as the kidney plays a crucial role in vascular regulation [13]. The relationship between obesity and cardiovascular conditions, including hypertension, vascular remodeling, atherosclerosis, and cardiac remodeling that results in dilated cardiomyopathy, are strongly medicated by renal function [3, 13, 16-17].

The benefits of weight loss through lifestyle interventions and bariatric surgery are well documented in terms of cardiovascular outcomes but have yet to be well established for renal function. Previous studies on bariatric surgery report improvements in glomerular filtration rate (GFR) ranging from 13-35 mL/min/1.73m^2^ and proteinuria from 0.06-0.53 grams/24 hours following surgical interventions [10, 18-20]. Despite the noted improvements associated with bariatric surgery, including the most significant amounts of weight loss, this form of treatment is only an option for a minority of patients. Bariatric surgery has limited accessibility due to cost, insurance coverage, strict guidelines based on BMI to be eligible for surgical intervention, availability of specialized surgical services including pre- and post-operative care, and patient concerns about potential short- and long-term complications and adverse effects. Data obtained from a study in 2017 by DeMaria et al. showed that approximately 1% of the population eligible for bariatric surgery underwent the procedure [21]. As a result, the majority of patients attempt weight loss through less invasive and more long-term interventions that focus on healthy eating and lifestyle changes. Dietary studies are often short term, narrowly focused on specific groups of participants or volunteers, and conducted in a structured clinical setting supported by multidisciplinary teams. As a result, the outcomes do not provide realistic guidance for patients and healthcare providers attempting to find the optimal weight loss strategy in a community setting, especially if resources are limited. The outcomes of these studies often focus on BMI and some studies address cardiovascular outcomes, but few have reported on the impact of weight loss through lifestyle interventions on renal function [10-11, 16, 22].

In order to better address the effects of a real-world, practical, community-based intervention for weight loss on renal function, a retrospective analysis was conducted of patients with obesity who participated in a community-based multimodal lifestyle intervention. Renal function was assessed with calculations of GFR at baseline and following 12 weeks of the intervention. Secondary outcomes included measurements of disease progression in hypertension, diabetes mellitus, and hyperlipidemia.

## Materials and methods

Research participants

Study participants were adults aged 18 years and older who voluntarily enrolled in a physician-directed, community-based weight management program between January 1, 2009 and March 31, 2014. All participants met the Health Management Resources (HMR) Program Medical Guideline criteria that excluded pregnant women, substance abusers, patients with eating disorders or aberrant eating behaviors, and patients who have been diagnosed with severe liver disease, renal failure, active malignancy, Cushing’s syndrome, bacterial endocarditis, osteomyelitis, or tuberculosis. Individuals were excluded from the analysis if they were receiving dialysis or if they failed to complete 12 weeks of the program. The study was approved by the Institutional Review Board (IRB) of Via Christi Hospitals, Incorporated, and by the Human Subjects Committee at the University of Kansas School of Medicine, Wichita (IRB Protocol Number: KU-VC 1656). All participants provided written informed consent prior to participation in the study.

Weight loss intervention

The weight loss program provides a comprehensive lifestyle modification approach to promote weight loss and maintenance of healthier lifestyles. Participants learn behavioral strategies to sustain a lower calorie diet through healthy eating, use of meal replacements, and to increase regular physical activity. Study participants are required to limit food choices to proprietary meal replacements (shakes, soups, cereal, and entrees) and to consume at least five meal replacements (shakes, soups, or cereal) along with two vitamin and mineral tablets per day. This regimen provided a minimum of 800 kilocalories per day as well as more than 100% of the recommended daily allowance of most vitamins and minerals. Participants were also encouraged to expend at least 300 kilocalories per day in planned physical activities. 

Participants met individually with behavioral and medical staff members prior to weekly group classes to report on compliance with the prescribed diet and to discuss any perceived barriers, exercise amounts, and medical concerns or changes in overall health. Medications for concurrent conditions, such as hypertension and diabetes mellitus, were adjusted by the physician weekly or as medically appropriate. Group classes were designed to provide support, accountability, and group problem solving regarding ways to overcome barriers to weight loss and to establish healthier lifestyles. Verified weights were recorded and entered into the clinical database every four weeks. Information on participant demographics and current medications was collected and verified from detailed medical histories and medical records provided by the participant’s primary care physician. 

Data collection

Study variables including weight, medication utilization, behavioral compliance, and laboratory studies were collected. The use of diabetic and anti-hypertensive medications was recorded at baseline, weekly, and at 12 weeks following the start of the intervention by self-report of the study participants and through a review of medical records from the participant’s primary care physician. Blood pressures were measured weekly to the nearest 2 millimeters mercury (mmHg) using an aneroid sphygmomanometer. Fasting blood specimens were collected for fasting plasma glucose, hemoglobin A1c (HbA1c), triglycerides, total cholesterol, high-density lipoprotein (HDL) cholesterol, calculated low-density lipoprotein (LDL) cholesterol, creatinine, and calculated GFR at baseline and immediately following 12 weeks from the start of the intervention. 

GFR was calculated from data collected upon initiation of the program and weekly for 12 weeks utilizing the CKD-EPI formula (GFR = 141 × min (Scr /κ, 1)α × max(Scr /κ, 1)-1.209 × 0.993Age × 1.018 [if female] × 1.159 [if black]). GFR calculations were used to classify patients into chronic kidney disease stage. Stage 1 CKD is classified by a GFR of ≥90 ml/min/1.73m^2^, Stage 2 with a GFR of 60-89 ml/min/1.73m^2^, Stage 3a with a GFR of 45-59 ml/min/1.73m^2^, Stage 3b with a GFR of 30-44 ml/min/1.73m^2^, Stage 4 with a GFR of 15-29 ml/min/1.73m^2^, and Stage 5 with a GFR of <15 ml/min/1.73m^2^ [23].

Heights were measured without shoes to the nearest 0.125 inch (0.32 centimeters). Verified body weights were measured to the nearest 0.1 pound (0.045 kilograms) with clothing and no shoes at baseline and at each weekly visit. BMI was calculated as the current mass in kilograms divided by the square of the current height in meters. 

Intake of meal replacements were self-reported to behavioral staff during weekly visits and verified by the purchase of replacement meals. Participants learned to calculate calories expended in physical activity based on intensity level, and the values were self-reported to behavioral staff during weekly visits. Other variables collected included participant age, gender, and race/ethnicity. Data were collected at baseline, following the 12-week lifestyle intervention, and at one-year post-intervention.

Statistical analysis

Statistical analyses were conducted by the authors using SAS Software for Windows (Version 9.3, Cary, NC). All data were analyzed on an aggregate level. Descriptive statistics were presented as means (M) and standard deviations (SD) for continuous variables and frequencies and percentages for categorical variables. Paired student t-tests were used to assess selected baseline and week 12 parameters including weight, BMI, GFR, CKD stage, creatinine levels, fasting plasma glucose, HbA1c, total cholesterol, triglycerides, HDL, LDL, and systolic and diastolic blood pressure. Chi-square analysis was conducted to assess the change in CKD stage at baseline and week 12. A correlation coefficient was calculated to assess the change in weight from baseline to week 12 and the change in GFR from baseline to week 12. All statistical tests were two-sided. A p-value ≤ 0.05 was considered statistically significant.

## Results

Of the 71 participants included in the final analysis, almost all were Caucasian (65: 91.6%) and two-thirds (45:63.4%) were women. The average age was 62.58 ± 9.31 years (range 38 to 80 years), and 48% of participants were over 65 years of age (Table 1). Approximately 73% of participants had class III obesity, 18% class II, 6% class I, and 3% were overweight. At baseline, 84% of participants had a diagnosis of hypertension and 63% had diagnosed diabetes mellitus. Of the 71 participants in the study, 92% were taking at least one anti-hypertensive medication and 60% used at least one medication to treat diabetes mellitus. Approximately 95 percent of participants in the study had tried multiple weight loss interventions with limited success prior to enrolling in the program. Participants within this study elected not to proceed with surgical interventions for a variety of reasons including cost, access to care, and concerns regarding surgical complications and adverse effects. 

**Table 1:** Participant demographic summary Demographics of participants that completed 12 weeks of the lifestyle intervention. Hypertension was defined as a blood pressure greater than 140/90 mmHg. Diabetes was defined as an HbA1c greater than 6.5%.

	Frequency (N=71)	Percent
Gender		
Male	26	36.60%
Female	45	63.40%
Ethnicity		
Caucasian	65	91.60%
Black	1	1.40%
Hispanic	2	2.80%
Other	3	4.20%
Age		
Less than 65	37	52.11%
65 and older	34	47.89%
Body Mass Index		
25-29.9	2	2.82%
30-34.9	4	5.63%
35.39.9	13	18.30%
greater than 40	52	73.25%
Diabetes Mellitus		
Yes	44	62.86%
No	26	37.14%
Hypertension		
Yes	60	84.51%
No	10	84.51%

The average weight at baseline was 289.38 ± 66.77 pounds (lbs), and weight continued to decrease at week 4 (279.95 ± 64.45 lbs), week 8 (268.31 ± 62.49 lbs) and week 12 (259.7 ± 60.72 lbs). The average weight loss of participants in the program was approximately 30 pounds in 12 weeks. The minimum amount of weight lost during the program was 23 pounds with a maximum amount of weight loss of 73 pounds. The patients with the greatest weight loss were in the 40-50s age group and lost the greatest amount of weight in the first four weeks of the program. Patients with the minimum amount of weight loss achieved their greatest amount of weight loss between weeks 4 and 8 with reductions in weight also noted during the first and last weeks of the program.

**Figure 1:**
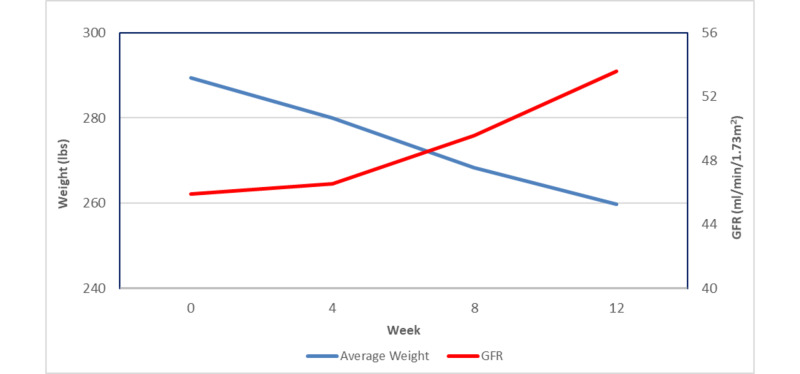
Correlation between weight loss and glomerular filtration rate A statistically significant correlation between weight loss and glomerular filtration rate (GFR) improvement was noted (correlation coefficient r= -0.4429, p= 0.0006). GFR values were reported in milliliters per minute per 1.73 meters squared (ml/min/1.73m^2^).  Weight is reported in pounds (lbs).

At baseline, the average GFR was 47.41 ± 10.11 ml/min/1.73m^2^ (Table 1). Approximately 58% (n=41) of participants were in stage 3A CKD, 32% (n=23) in stage 3B, and one participant had stage 4 CKD at baseline (Table 2). Following the completion of the 12-week intervention, 76% of participants (n=51) had improved renal function. The average GFR significantly improved by 7.76 ± 13.05 ml/min/1.73m^2^ from baseline (p<0.0001). Only 23% of participants (n=15) remained in the same CKD stage (Table 2). The most frequent CKD stage at week 12 was stage 2 (35.8%, n=24), followed by stage 1 (22.9%, n=20) (Table 1). A highly statistically significant correlation was found (correlation coefficient r= -0.4429, p= 0.0006) between weight loss and GFR improvement (Figure 1). The greatest improvements in GFR were noted within the first four weeks of weight loss with more significant improvements in GFR noted in participants who lost the most weight.

**Table 2:** Comparison of baseline chronic kidney disease stage with week 12 chronic kidney disease stage Overall, 76.1% (51 of 67) of participants decreased in stage, 22.4% (15 of 67) remained within the same stage, and 1.5% (1 of 67) progressed from stage 3A to 3B. Four participants did not have a week 12 chronic kidney disease (CKD) stage and were excluded from the analysis.

		Week 12 Chronic Kidney Disease Stage				
		1	2	3A	3B	4
Baseline Chronic Kidney Disease Stage	2A	0 (0%)	1 (100%)	0 (0%)	0 (0%)	0 (0%)
	3A	14 (34.2%)	18 (43.9%)	8 (19.5%)	1 (2.4%)	0 (0%)
	3B	5 (25%)	5 (25%)	4 (20%)	6 (30%)	0 (0%)
	4	1 (20%)	0 (0%)	0 (0%)	3 (60%)	1 (20%)

Secondary outcome measures, including fasting blood sugar, HbA1c, total cholesterol, triglycerides, LDL, and systolic and diastolic blood pressures, showed statistically significant improvement following 12 weeks of therapy (Table 3). Fasting plasma glucose decreased on average by 16 milligrams per deciliter (mg/dL) at week 12 (p=0.0542) and HbA1c decreased from 7.7% at baseline to 6.7% at week 12 (p=0.0012). Lipid profiles improved following 12 weeks of the intervention with cholesterol decreasing on average by 26 mg/dL (p<0.0001), LDL decreased by 15 mg/dL (p=0.0041), and triglycerides decreased by 34 mg/dL (p=0.0012). HDL was the only clinical measure that did not show a significant improvement from baseline to week 12. Blood pressure was noted to decrease from an average of 130/72 mmHg to 121/68 mmHg following the weight loss intervention (p=0.0002 for systolic blood pressure (SBP) and p=0.0253 for diastolic blood pressure (DBP)). A positive correlation of 0.16 was noted between improvements in SBP and GFR following 12 weeks of therapy (p=0.2215).

**Table 3:** Secondary outcome comparisons between baseline and week 12 All values were presented as mean ± standard deviation. Fasting blood sugar, total cholesterol, low-density lipoprotein (LDL), high-density lipoprotein (HDL), and triglycerides were all reported as milligrams per deciliter (mg/dL). Hemoglobin A1c (HbA1c)  is the representation of the amount of glycated hemoglobin in the blood and is presented as a percentage. Systolic and diastolic blood pressure are presented in millimeters mercury (mmHg). Weight is reported in pounds (lbs). Glomerular filtration rate (GFR) is reported in milliliters per minute per 1.73 meters squared (ml/min/1.73m^2^).

Clinical Outcomes	Baseline	Week 12	Change from Baseline	P-values
Fasting Blood Sugar (mg/dL)	129.76 ± 59.57	113.36 ± 32.47	-16.41 ± 64.12	0.0542
HbA1c	7.71 ± 1.73	6.69 ± 1.12	-1.03 ± 1.35	0.0012
Cholesterol (mg/dL)	176.12 ± 42.19	150.11 ± 32.9	-26.02 ± 43.65	<0.0001
Systolic Blood Pressure (mmHg)	129.70 ± 15.74	121.17 ± 14.33	-8.53 ± 16.71	0.0002
Diastolic Blood Pressure (mmHg)	72.53 ± 11.53	68.53 ± 9.56	-4.00 ± 13.5	0.0253
LDL (mg/dL)	91.27 ± 35.19	76.07 ± 27.92	-15.20 ± 37.21	0.0041
HDL (mg/dL)	46.47 ± 15.05	42.95 ± 26.38	-3.53 ± 22.69	0.2456
Triglycerides (mg/dL)	193.70 ± 99.56	159.30 ± 71.43	-34.40 ± 76.03	0.0012
Weight (lbs)	289.38 ± 66.77	259.70 ± 60.72	-29.68 ± 22.09	<0.0001
GFR (ml/min/1.73m^2^)	47.41 ± 10.11	55.17 ± 17.03	7.76 ± 13.05	<0.0001

In subgroup analysis, non-diabetic patients had significantly greater weight loss than diabetic patients with a 37.43 lb change from baseline in non-diabetics and 24.86 lb change from baseline in diabetics (p=0.0211) (Table 4). No significant differences were found in changes of GFR following weight loss at -5.89 ml/min/1.73m^2^ and -11.41 ml/min/1.73m^2^ in diabetics and non-diabetics respectively (p=0.1429). Additionally, no significant differences were noted in clinical outcomes (all p-values>0.05).

**Table 4:** Secondary outcome comparisons between baseline and week 12 in diabetics and non-diabetics P-value was based on the test of the change of clinical outcomes (baseline less week 12) between diabetic and non-diabetic populations. All values were presented as mean ± standard deviation. Fasting blood sugar, total cholesterol, low-density lipoprotein (LDL), high-density lipoprotein (HDL), and triglycerides were all reported as milligrams per deciliter (mg/dL). Hemoglobin A1c (HbA1c) represents the amount of glycated hemoglobin in the blood and is presented as a percentage. Systolic and diastolic blood pressure are presented in millimeters mercury (mmHg). Weight is reported in pounds (lbs). Glomerular filtration rate (GFR) is reported in milliliters per minute per 1.73 meters squared (ml/min/1.73m^2^).

	Non-Diabetics			Diabetics			
Clinical Outcomes	Baseline	Week 12	Change from Baseline	Baseline	Week 12	Change from Baseline	P-values
Fasting Blood Sugar (mg/dL)	107.91 ± 17.28	98.59 ± 15.63	9.32 ± 15.59	138.72 ± 67.85	121.69 ± 37.08	17.03 ± 78.27	0.6517
HbA1c	--	--	--	7.75 ± 1.76	6.73 ± 1.13	1.03 ± 1.38	--
Systolic Blood Pressure (mmHG)	125.55 ± 15.59	119.36 ± 12.95	6.18 ± 15.64	132.22 ± 15.73	122.7 ± 15.04	9.51 ± 17.44	0.7755
Diastolic Blood Pressure (mmHG)	72.86 ± 10.73	69.36 ± 9.55	3.5 ± 17.35	72.41 ± 12.26	68.27 ± 9.7	4.14 ± 11.05	0.6926
Cholesterol (mg/dL)	187.75 ± 51.24	147.2 ± 37.46	40.55 ± 65.7	170.78 ± 35.63	152.69 ± 30.39	18.08 ± 22.77	0.2515
LDL (mg/dL)	100.58 ± 41.94	74.95 ± 30.36	25.63 ± 55.71	86.64 ± 30.77	77.41 ± 26.99	9.22 ± 20.74	0.4352
HDL (mg/dL)	44.9 ± 9.98	37.35 ± 7.51	-7.55 ± 6.46	47.83 ± 17.21	46.39 ± 32.38	-1.44 ± 28.04	0.3230
Triglycerides (mg/dL)	213.6 ± 109.88	169.9 ± 89.72	43.7 ± 83.21	183.19 ± 94.63	153.33 ± 60.76	29.86 ± 73.52	0.8772
Weight (lbs)	290.93 ± 85.58	253.5 ± 74.42	37.43 ± 15.87	288.41 ± 53.18	263.55 ± 51.18	24.86 ± 24.16	0.0211
GFR (ml/min/1.73m^2^)	52.59 ± 5.52	64 ± 16.57	-11.41 ± 13.86	44.22 ± 11.12	50.11 ± 15.38	-5.89 ± 12.27	0.1429

Following 12 weeks of the intervention, 64% of participants required fewer anti-hypertensive medications and 83% required fewer medications to treat diabetes mellitus. The overall patient cost of the weight loss program was approximately $200 per week for classes and $110 per week for meal replacements. Eighty percent of participants in the study reported an overall weekly cost savings associated with the program due to reduced costs associated with grocery shopping and eating outside of the home. These reductions resulted in a significant overall cost savings to the patient and healthcare system. 

Compliance with the program was high among participants with approximately 85% of participants adhering to the meal replacements and prescribed weekly exercise expenditures. All participants included in the study completed the 12-week intervention. At one-year post-intervention, approximately 49% of patients were lost to follow up, which was attributed to participants moving out of the region, meeting weight loss goals, loss to follow up due to weight gain, or pursuing additional mechanisms to achieve weight loss. Of the 35 participants in the one-year post-intervention follow up, 89% maintained their weight or continued to lose weight.

## Discussion

This study verifies that substantial weight reduction and associated improvements in clinically significant outcomes are achievable through community-based programs that focus on nutrition and behavioral change. Results demonstrate a strong positive correlation between weight loss and improvements in renal function following a 12-week program of intensive lifestyle interventions. Significant improvements were also noted in fasting blood sugar levels, HbA1c, lipid parameters, and blood pressure readings following weight loss. While the weight loss results achieved in this study are not as dramatic as those reported following bariatric surgery, the reductions obtained from this program are sufficient to have a meaningful impact on renal outcomes and other markers of chronic disease. On a population basis, small weight reductions achieved by large numbers of individuals are more meaningful than large weight losses achieved by fewer individuals using medically or surgically intensive interventions.

Participants lost on average 30 pounds and showed significant improvement in GFR. This finding correlates with previous results showing that weight loss of an average of 10 kg through a combination of dietary modification and exercise resulted in an 18-point improvement in GFR [24]. Participants who lost the most weight in the first four weeks of the program recorded their most significant improvements in renal function during this timeframe. This result reflects greater adherence to the lifestyle changes in the initial phases of the intervention, but the overall reductions in caloric intake and increased energy expenditures throughout the study may have resulted in sustained improved metabolic parameters and greater renal blood flow. Previous studies have shown improved renal hemodynamics following weight loss, including increased renal profusion and decreased systemic hypertension [25]. Weight loss has also been shown to reduce microalbuminuria and proteinuria that can lead to glomerular basement membrane damage and fibrosis [3, 26-27]. These positive hemodynamic changes of weight loss have the potential to reduce or delay the progression of glomerulonephropathy in obese patients and improve overall renovascular and cardiovascular risk factors. The positive effect between weight loss and GFR persisted when controlling for diabetes and hypertension suggesting that the improvement in renal function is independent of additional obesity-related chronic diseases. This effect has been observed in previous studies where the relationship between improvements in BMI and CKD persisted after controlling for the independent risk factors of diabetes and hypertension [28-30].

The cost of the program and access to resources, such as group meetings, can be limiting to participants, especially those in living in rural areas. The characteristics of the program, especially the “operator effect” of individual counselors limits the generalizability of the results. Nevertheless, similar programs could be implemented including the use of telemedicine or online interventions that allow participants in remote locations to participate in group coaching sessions. A prospective, long term study of at least 5 to 10 years post intervention would be needed to determine if the effects of weight loss on GFR and improvement in CKD stage persisted with continued healthy lifestyle changes.

This study is limited in generalizability due to small sample size, the study being conducted at a single location, and the demographics of participants in the study. The study population was largely Caucasian with a female predominance. Additional studies that address diverse populations with regards to cultural beliefs surrounding weight loss and obesity would be needed to further explore the application of a community-based weight loss program in diverse populations. Additionally, studies have shown that the Hispanic and African American communities, which were underrepresented in this study (one and two patients respectively), have a disproportionate burden of obesity affecting 49% of the African American population and 45% of the Hispanic population [2]. A long-term lifestyle-based weight loss intervention study that focuses on these communities with a larger sample size at diverse study sites would be needed to better address the impact of obesity and weight loss interventions within these high-risk communities.

Results obtained from this study suggest that the impact of weight loss on chronic kidney disease plays a significant role in reducing renovascular and subsequently cardiovascular outcomes by optimizing risk factors including blood sugar levels, lipid parameters, and blood pressure through lifestyle interventions. The etiology of the improvement in renal function observed in participants in this study could not be determined due to the retrospective design of the project. Additional long-term prospective studies including a large sample size conducted at numerous study sites on the mechanisms underlying the improvement in renal function will be needed to fully appreciate the positive impact of weight loss on overall renovascular health for individuals and on a population level. Such studies are likely to focus on the role of inflammation, hemodynamic effects of weight loss, oxidative stress, and the degree of proteinuria improvement resulting in decreased renal fibrosis. Additionally, prospective studies will need to consider the generalizability of study results, ways to enhance participant compliance, and address barriers to implementation including limited access to resources and cultural factors that can impede weight loss.

## Conclusions

In conclusion, this study demonstrated that significant reductions in body mass, achieved through a 12-week community-based multidisciplinary lifestyle modification program, resulted in improved renal function and prevention of chronic kidney disease progression in individuals with obesity. Weight loss achieved through dietary modification, behavioral change, and increased energy expenditures resulted in an improvement in markers for chronic diseases including diabetes mellitus, hypertension, and hyperlipidemia. Optimization of risk factors through weight loss achieved by lifestyle modification programs can improve overall renovascular and cardiovascular outcomes for patients with obesity resulting in overall reductions in obesity-related morbidity and mortality.
